# The gut–brain axis in early Parkinson’s disease: from prodrome to prevention

**DOI:** 10.1007/s00415-025-13138-5

**Published:** 2025-05-21

**Authors:** Patrick James Oliver, Livia Civitelli, Michele T. Hu

**Affiliations:** 1 Clinical Medical School, University of Oxford, Oxford, UK; 2 Green Templeton College, University of Oxford, Oxford, UK; 3 Nuffield Department of Clinical Neurosciences, Oxford Parkinsons’ Disease Center, University of Oxford, Oxford, UK; 4 Oxford Parkinson’s Disease Centre, University of Oxford, Oxford, UK; 5 Nuffield Department of Clinical Neurosciences, University of Oxford, Oxford, UK; 6 Department of Neurology, West Wing, Level 3, John Radcliffe Hospital, Headley Way, Oxford, OX3 9DU UK

**Keywords:** Parkinson’s disease, α-Synuclein, Microbiome, Gut–brain axis, Rapid eye movement sleep behavior disorder (RBD), Prodromal

## Abstract

Parkinson’s disease is the second most common neurodegenerative disorder and fastest growing neurological condition worldwide, yet its etiology and progression remain poorly understood. This disorder is characterized pathologically by the prion-like spread of misfolded neuronal alpha-synuclein proteins in specific brain regions leading to Lewy body formation, neurodegeneration, and progressive neurological impairment. It is unclear what triggers Parkinson’s and where α-synuclein protein aggregation begins, although proposed induction sites include the olfactory bulb and dorsal motor nucleus of the vagus nerve. Within the last 20 years, there has been increasing evidence that Parkinson’s could be triggered by early microbiome changes and α-synuclein accumulation in the gastrointestinal system. Gut microbiota dysbiosis that alters gastrointestinal motility, permeability, and inflammation could enable prion-like spread of α-synuclein from the gut-to-brain via the enteric nervous system. Individuals with isolated rapid eye movement sleep behavior disorder have a high likelihood of developing Parkinson’s and might represent a prodromal ‘gut-first’ subtype of the condition. The gut-first model of Parkinson’s offers novel gut-based therapeutic avenues, such as anti-, pre-, and pro-biotic preparations and fecal microbiota transplants. Crucially, gut-based interventions offer an avenue to treat Parkinson’s at early prodromal stages with the aim of mitigating evolution to clinically recognizable Parkinson’s disease characterized by motor impairment.

In his 1817 seminal essay, James Parkinson suggested that ‘The Shaking Palsy’ arises from a “disordered state of the stomach and bowels” [1]. Parkinson suggested this gastrointestinal disturbance was responsible for the slowing of movements, rest tremor, and rigidity seen in ‘The Shaking Palsy’ which was renamed as Parkinson’s disease (PD) [2]. In the two centuries following Parkinson’s work, several seminal studies suggested the pathophysiology of PD originated in the brain. In the mid-20^th century^, Carlsson identified that injections of the dopamine precursor levodopa could restore motor activity in rabbits that had been rendered catatonic [3] After this, researchers in 1961 showed that intravenous injections of levodopa to patients with PD reduced dopaminergic deficit and provided symptom control [4]; while in contrast toxin-induced degradation of nigrostriatal dopaminergic neurons was repeatedly shown to induce PD-like motor deficits in animal models throughout the 20th century [5]. Detailed neuropathological studies have characterized the temporal progression of α-synuclein aggregates that are found in PD that cause neurodegeneration, providing further support for a so-called brain-first model of the disease [6]. In this model outlined by Braak et al., early α-synuclein induction sites are found in the brain, including the dorsal motor nucleus or the vagus and the olfactory bulb, with spread to other brain regions and peripheral non-brain sites only occurring at late stages of the disease [7, 8].

Recent evidence has suggested a return to Parkinson’s early hypothesis and a paradigm shift in our understanding of PD: namely, that PD begins in the stomach and bowels. This is encapsulated by the gut-first model of PD where microbiome–gut–brain axis dysfunction is central to disease pathogenesis [9]. First, non-motor symptoms such as constipation and bloating are among the earliest seen in prodromal PD [10, 11]. Second, there is increasing evidence that α-synuclein aggregates are found outside the brain in PD, before the onset of motor symptoms [12–14]. Third, cellular and animal studies have provided a model for prion-like spread of α-synuclein from the gut to the brain via the enteric nervous system, chiefly through the vagus nerve [15–17]. It has been shown that alterations in the various bacteria, fungi, viruses, and other biological kingdoms that make up the gut microbiome are associated with PD [18]. Currently, it is unclear whether microbiome changes are specific to PD, and whether they cause, contribute to, or follow disease progression. The most popular model of gut-first PD is that changes in the gut microbiome and its metabolites causes local inflammation and increased gut luminal permeability, enabling permissive aggregation and seeding of α-synuclein that can spread to the brain via the vagus nerve [9, 19]

Rapid eye movement sleep behavior disorder (RBD) is associated with a high likelihood of developing PD and individuals with RBD show alterations in the gut microbiome [20, 21]. This review will synthesize available evidence to consider whether RBD could represent a prototypical gut-first subtype of PD. This offers hope for developing early gut-based biomarkers to identify prodromal PD and gut-based therapeutic interventions to prevent phenoconversion: progression of prodromal PD characterized by RBD to PD associated with clinical motor symptoms of bradykinesia, rest tremor, and ataxia. The central question of this review is whether a gut-based understanding of PD aid its earlier identification and treatment with novel gut-based interventions? The hope is that gut-based treatments can complement existing treatments, improve both motor- and non-motor symptom control, and attenuate the progression of prodromal PD to late disease stages marked by severe motor and functional impairment.

## Prodromal PD is a non-motor phenomenon

Prodromal PD, characterized by the initiation of neurodegeneration before the clinical signs of PD are evident, mostly comprises non-motor symptoms [22]. James Parkinson identified constipation as a common feature in individuals with the shaking palsy that preceded the onset of motor symptoms. Recent analyses reveal that early constipation and gastrointestinal complaints are seen in 80% of PD patients [23, 24]. Other studies have linked leucine-rich repeat kinase 2 mutations (which are a cause of monogenic autosomal dominant PD) to inflammatory bowel diseases (IBDs) that are characterized by gastrointestinal barrier dysfunction. Large data studies have revealed an elevated risk of PD in individuals with IBDs such as Crohn’s disease and ulcerative colitis [25, 26], while anti-tumor necrosis factor treatments that decrease gastrointestinal inflammation and improve intestinal permeability dysfunction can reduce the risk of developing PD [27]. Recent evidence has also pointed towards shared changes in the gut microbiome in PD and IBD [28]. A leaky gut barrier has been linked to PD pathogenesis involving local flux of bacterial toxins, microbes, and potentially molecules like α-synuclein across the gastrointestinal epithelium [29–31]; while systemically gut inflammation drives pro-inflammatory cytokine production, blood–brain barrier disruption, and migration of inflammatory cells into the brain causing neuronal dysfunction and cell death [32, 33]. Other non-motor symptoms seen in prodromal PD include sleep disturbances, depression, anxiety, and hyposmia, although a range of non-motor symptoms continue to manifest even after a clinical diagnosis of PD is made, such as autonomic dysfunction (urinary symptoms and orthostatic hypotension), fatigue, apathy, pain, mild-cognitive impairment, and ultimately dementia [34, 35]. Strikingly, non-motor symptoms remain poorly responsive to current PD treatments despite being present from early prodromal PD and contributing significantly to overall disease burden [36]. Prodromal PD represents a window for earlier disease identification and intervention, as well as highlighting that PD has many non-motor complaints that are often overlooked.

Sleep disturbances are closely linked to prodromal PD. In the 1970s, Michel Jouvet used brainstem lesions in cats to identify the neuroanatomical sites responsible for muscle atonia during rapid eye movement (REM) sleep [37]. Then, in 1986, Carlos Schenck identified the seminal link between idiopathic REM sleep behavior disorder (RBD) and PD [20]. RBD is characterized by loss of muscle atonia during sleep, resulting in affected individuals acting out their dreams: characteristic features include hitting, kicking, and even attempted strangulation with vocalizations including shouting, screaming, and laughing [38]. Clinically, RBD is identified through polysomnographic sleep recordings. Epidemiological studies have shown that RBD is common in the prodromal stages of α-synucleinopathies—PD, dementia with Lewy bodies, and multiple systems atrophy—while remaining rare in other neurodegenerative conditions, such as tauopathies and amyloidopathies [39]. This is probably because α-synucleinopathies are associated with cell loss in the neuronal structures regulating REM sleep atonia, which animal studies have localized to the pontine sub-coeruleus and central medulla of the brainstem, thought to contain a putative ‘flip-flop’ switch for REM sleep control [40, 41]. The largest study to date of 1280 polysomnographically diagnosed RBD subjects from 24 international RBD study group sleep centers found an overall phenoconversion rate from RBD to overt α-synucleinopathies of 6.3% per year, with 73.5% converting after 12-year follow up. This shows that RBD is a strong indicator that somebody will develop an α-synucleinopathy, most commonly PD.

**Fig. 1:**
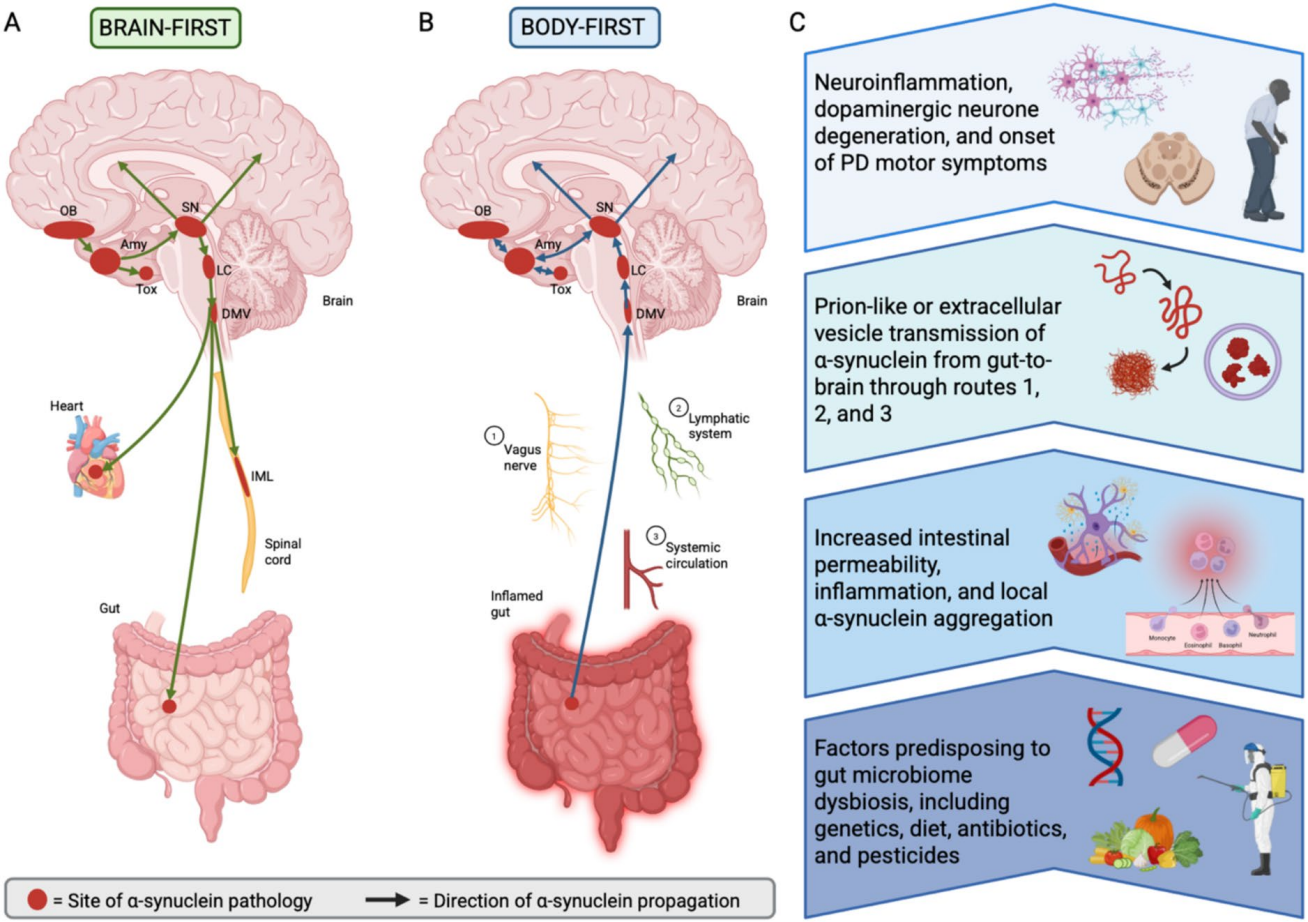
Temporal pathogenesis of brain- and body-first PD subtypes. A. In brain-first PD, α-synuclein aggregates are initially found in the olfactory bulb and propagate towards the substantia nigra before spreading to the rest of the body. B. In body-first PD, prodromal symptoms of constipation, RBD, orthostatic hypotension, and bladder problems occur up to 20 years before aggregates spread upwards to affect the dopaminergic neurons of the brainstem substantia nigra. This spread is thought to involve the vagus nerve (1), lymphatic system (2), and systemic circulation (3) to varying extents, utilizing mechanisms such as prion-like propagation and extracellular vesicles. C. Flowchart illustration of the pathophysiological sequence from gut dysbiosis to PD in body-first subtypes, identifying key events in disease initiation and progression. Amy, amygdala; DMV, dorsal motor nucleus of vagus; IML, intermediolateral column; LC, locus coeruleus; OB, olfactory bulb; SN, substantia nigra; ST, sympathetic trunk; Tox, transentorhinal cortex. (Created with BioRender.com)

Around 50% of the individuals with PD will not have RBD at any stage of their condition, leading to the hypothesis that RBD-positive PD might represent a distinct subtype of the condition. In support, RBD-positive cases of PD are associated with faster progression and worse prognosis, suggesting distinct pathophysiology from RBD-negative cases [42]. Based on peripheral radiographic scintigraphy studies and radiographic DOPA positron emission tomography from the brain, it is speculated that RBD-positive PD is associated with early degeneration of cardiac and gastrointestinal autonomic innervation with nigrostriatal dopamine sparing [43, 44]. These findings would be consistent with initiation of pathology outside the brain (bottom up). Similar imaging studies show that RBD-negative patients have predominantly intact cardiac autonomic innervation, potentially explained by initiation of the disease in the brain (top down). The combination of imaging and correlative clinical findings suggest that RBD-positive PD might correspond to a body-first disease phenotype, while RBD-negative PD better represents brain-first disease progression (Fig. 1) [45, 46]. These subtypes might also be distinguished by body-first PD involving a longer prodrome, more dysautonomia and symmetrical parkinsonism, faster progression, and higher dementia risk [47, 48]. Further possible classifications of prodromal PD will not be considered further in this review, such as genetic and biological subtypes [49]. The theory that RBD-positive PD represents a distinct subtype with bottom-up pathogenesis involving earlier gastrointestinal and autonomic disturbances remains a working hypothesis, currently with supportive evidence from clinical imaging studies but little support from cellular or histopathological analysis of gut and autonomic tissues in humans. If this hypothesis is correct, it will influence the subset of prodromal PD patients which will be suitable for microbiome-based investigations and treatments to prevent phenoconversion, as discussed later in this review [50].

## α-Synuclein aggregates are found outside the brain in PD

It is unclear whether PD begins inside or outside the brain. Conflicting evidence has come from a mixture of epidemiological, animal, autopsy, and imaging studies. Braak et al. proposed a dual-hit hypothesis for PD in 2007 postulating that a neurotropic pathogen simultaneously triggered α-synuclein aggregation in the olfactory bulb and dorsal motor nucleus of the vagus (DMV) which could then further invade the brain from these two entry points [51, 52]. However, a recent revision based on post-mortem analysis of many patients with mild Lewy body disease including PD and other related α-synucleinopathies, suggests that α-synuclein pathology typically originates in either the olfactory bulb or the peripheral nervous system (including the DMV), but rarely both simultaneously [53]. The dual-hit hypothesis outlines how a nasal route for a neurotropic pathogen would involve anterograde progression to the temporal lobe and olfactory bulb, while spread to the DMV would rely on a neurotropic pathogen from a gastric route. One possible conclusion is that brain- and body-first PD could have nasal and gastric sources, respectively.

Retrograde spread of α-synuclein pathology from a gastric source to the DMV is thought to involve the vagus nerve [54, 55]. The most popular model for this theory is that α-synuclein spreads via coeliac and mesenteric ganglia from the gastrointestinal tract to sympathetic and parasympathetic trunks, including the heart, stellate ganglion, and intermediolateral column of the spinal cord [56]. This proposition gained mixed support from two large epidemiological studies showing that full truncal but not selective vagotomy decreases the risk of PD [57, 58]. The discrepancy in results could be attributed to the mixed effects of vagotomy: full truncal might prevent peripheral-to-central propagation of α-synuclein by completely removing the conduit for transport, while the effects of reduced α-synuclein transport in selective vagotomy are ameliorated by triggering a pro-inflammatory response that promotes pathological α-synuclein aggregation through disruption of the anti-inflammatory cholinergic pathways in the vagus nerve [59, 60]. Another explanation for the discrepancy could be that if body-first and brain-first PD truly represent distinct subtypes of the condition, the effect size of epidemiological studies would be reduced by mixing subtypes where presumed RBD-negative brain-first PD patients would not expect to gain a protective effect from vagotomy. Overall, vagotomy studies provide uncertain evidence for gut-to-brain propagation of α-synuclein in PD.

There is conflicting evidence in support of α-synuclein being detected peripherally before in the brain of PD patients. Since the 1960s, multiple post-mortems and immunohistochemical studies have identified α-synuclein in the esophagus, stomach, small intestine, and enteric nervous system up to 20 years before PD diagnosis [61, 62]. This could explain the presence of peripheral non-motor symptoms in prodromal PD. Despite this, a large study by the Arizona Parkinson’s consortium group did not find a single case in which α-synuclein was present in the enteric nervous system but not in the central nervous system in a survey of whole-body autopsies from individuals with Lewy body disorders (including PD) and matched controls [63]. There are several limitations of this survey which represent more general issues in the field of α-synuclein research, including: small samples from a large gastrointestinal tract might miss highly localized α-synuclein deposits; α-synuclein spread from gut-to-brain could occur rapidly and be missed by the poor temporal sensitivity of post-mortem analysis; and large heterogeneity in the forms of α-synuclein that exist in the body and techniques used to investigate this [64, 65]. For example, the Arizona consortium used a pS129 antibody which binds to phosphorylated α-synuclein, yet α-synuclein accumulation is preceded by aggregation steps before phosphorylation takes place which could be missed by pS129 antibody studies [66]. Future studies using antibodies that can detect immature, un-phosphorylated forms of α-synuclein found outside the brain will help us to understand where α-synuclein aggregation begins in prodromal PD [67] In summary, lesional and histopathology-based studies provide moderately supportive evidence for the gut-first model of PD, albeit with several inconsistencies between the existing established evidence. It remains unclear whether these discrepancies are due to methodological issues and differences between study protocols, or whether the currently theoretical gut-first model is incorrect—or perhaps requires revision. It is possible for each of these interpretations to be true and further studies will help to resolve these outstanding questions.

Advances in alternative techniques for α-synuclein analysis will help to resolve the temporal profile of PD pathogenesis. The low levels of α-synuclein isolated from gastrointestinal biopsies could be improved by using ultrasensitive techniques like seed amplification assay (SAA) that amplifies aggregated α-synuclein [68–70]. SAA has already proven to be a robust technique for identifying pathological α-synuclein from cerebrospinal fluid sample from patients with PD and RBD [71, 72]. The field is also moving toward assessing other biofluids, such as sebum from the skin, blood, gut biopsy, and fecal samples, which are less invasive than collecting cerebrospinal fluid samples [73–76]. There are other more novel techniques available for analyzing peripheral α-synuclein, such as two-dimensional electrophoresis, mass spectrometry, and α-synuclein specific ligands which can be detected via PET scans, although these methods are less well evidenced and won’t be discussed further in this review [6, 77]. Improvements in non-invasive techniques for identifying peripheral α-synuclein through skin, blood, and fecal samples could aid identification of prodromal PD, useful as a disease biomarker and enabling earlier therapeutic intervention. In addition, utilizing these techniques could help to determine whether the differences in findings between current established evidence is due to methodological or conceptual flaws in the gut-first model of PD.

## Prodromal PD might involve prionoid gut-to-brain propagation of α-synuclein

If α-synuclein aggregation truly begins in the periphery, how does it get to the brain to cause the motor symptoms seen in PD? One leading and provocative model suggests that it occurs through prion-like propagation: α-synuclein is transported intra-axonally, released into the extracellular space, taken up by neighboring neurons, where it seeds aggregation and endogenous α-synuclein synthesis inside the next cell [54, 78]. This draws parallels to the process of central nervous system invasion in other neurological prion disorders like variant Creutzfeldt Jacob Disease and bovine spongiform encephalopathy [77]. In 2008, Steiner et al. documented α-synuclein Lewy body pathology in grafted neurons in people with PD who had been transplanted over a decade prior to autopsy. The group suggested that α-synuclein in the grafted neurons was the result of permissive templating and prion-like spread of α-synuclein pathology from neurons in the host to those in the grafts [79]. Similarly, a controversial paper by Collinge et al. in 2024 suggested that certain rare-forms of Alzheimer’s disease are underpinned by prion-like seeding of amyloid proteins [80]. Therefore, PD might be one of many neurological disorders involving prion protein aggregation and propagation [81]. Other models of gut-to-brain spread of α-synuclein have been proposed, including circulatory, humoral, and lymphoid immune-mediated pathways [82] (Fig. 1), potentially involving extracellular vesicles to varying extents; since these potential mechanism of spread are less-well evidenced they will not be discussed further in this review.

Several studies have found support for prionoid propagation of α-synuclein from gut-to-brain. Prionoid propagation describes where protein aggregates display cycles of nucleation, fragmentation, and propagation, like prions, without evidence of inter-individual transmissibility [83, 84]. In 2016, Breid et al. performed a single intraperitoneal injection of α-synuclein fibrils prepared from *Escherichia coli* into five α-synuclein over-expressing mice [85]. Four developed paralysis and α-synuclein pathology in the CNS, including the substantia nigra, suggesting gut-to-brain α-synuclein propagation which the authors posited occurred in a prion-like manner. Despite this, Breid’s study had a very small sample size, used an α-synuclein over-expressing model of PD which doesn’t accurately capture the genotype of most PD patients (only 5–10% of PD patients have mutations in genes like SCNA, which encodes α-synuclein) [2], and was subject to a confirmation bias because α-synuclein spread is more likely to be observed in α-synuclein over-expressing mice. In summary, although prionoid gut-to-brain propagation of α-synuclein is proven to be possible in an artificial lab setting, further studies are required to elucidate this process in prodromal PD and provide proof of concept.

Given that not all humans or animal models displaying peripheral α-synuclein aggregation develop PD, there must be other factors which regulate gut-to-brain α-synuclein propagation. Most animal models investigating prionoid propagation of α-synuclein exhibit age-dependent effects. For example, Breid’s study was not successfully replicated in young wild-type rats or young non-human primates [45]. Since age is the greatest risk factor for PD, it is conceivable that young animals, or humans for that matter, can mount a sufficient defense against a peripheral α-synuclein insult that minimizes or completely prevents neuro-invasion. Another key factor modulating the ability of α-synuclein to initiate PD pathology is the gut microbiome. A study of Fischer-344 wild-type rats, which spontaneously develop asymptomatic α-synuclein pathology in the enteric nervous system in old age, showed that they will begin to develop pathological α-synuclein deposits in the brain and parkinsonian motor symptoms when their microbiome is disturbed through introduction of *Escherichia coli* [86, 87]. A recent study of transgenic α-synuclein overexpressing mice using gut microbial shotgun metagenomics highlighted that the onset of PD pathology is related to changes in gut bacterial taxa in an age- and genotype-dependent manner [88]. Since gastrointestinal symptoms feature heavily in prodromal PD, it is thought that changes in gut health could create a permissive microenvironment for gut-to-brain α-synuclein propagation.

## Gut microbiota dysbiosis is seen in prodromal PD

The human gastrointestinal microbiome consists of bacteria, archaea, protists, fungi, their respective viruses, and human viruses [77]. The number of genes encoded in the gut metagenome is approximately 150 times larger than that of the human genome [89]. Gut micro-organisms are crucial for nervous system functions such as myelination, neurogenesis, microglial activation, and modulating cognition and behavior; gut micro-organisms are also increasingly implicated in modifying susceptibility to and progression of neurodegenerative diseases, including PD [90, 91]. For example, one study showed that when α-synuclein over-expressing transgenic mice were raised in a germ-free environment they rarely developed the cardinal features of PD such as rest tremor, bradykinesia, and rigidity. When these mice were either colonized with feces from wild-type mice or orally fed bacterial metabolites like short chain fatty acids (SCFAs) without colonization, they developed a PD-like phenotype [92]. These findings were replicated with fecal transfer from humans with PD, but not with healthy age-matched controls. Gut dysbiosis involves an altered composition of the organisms found in the gut microbiome, such as a loss of normal healthy commensals, an overgrowth of pathobionts which have the potential to cause pathology, or a decrease in microbial species diversity [93, 94]. In synthesis, it is proposed that gut microbiota dysbiosis drives unhealthy disease states that enable permissive seeding of α-synuclein from gut-to-brain.

**Fig. 2:**
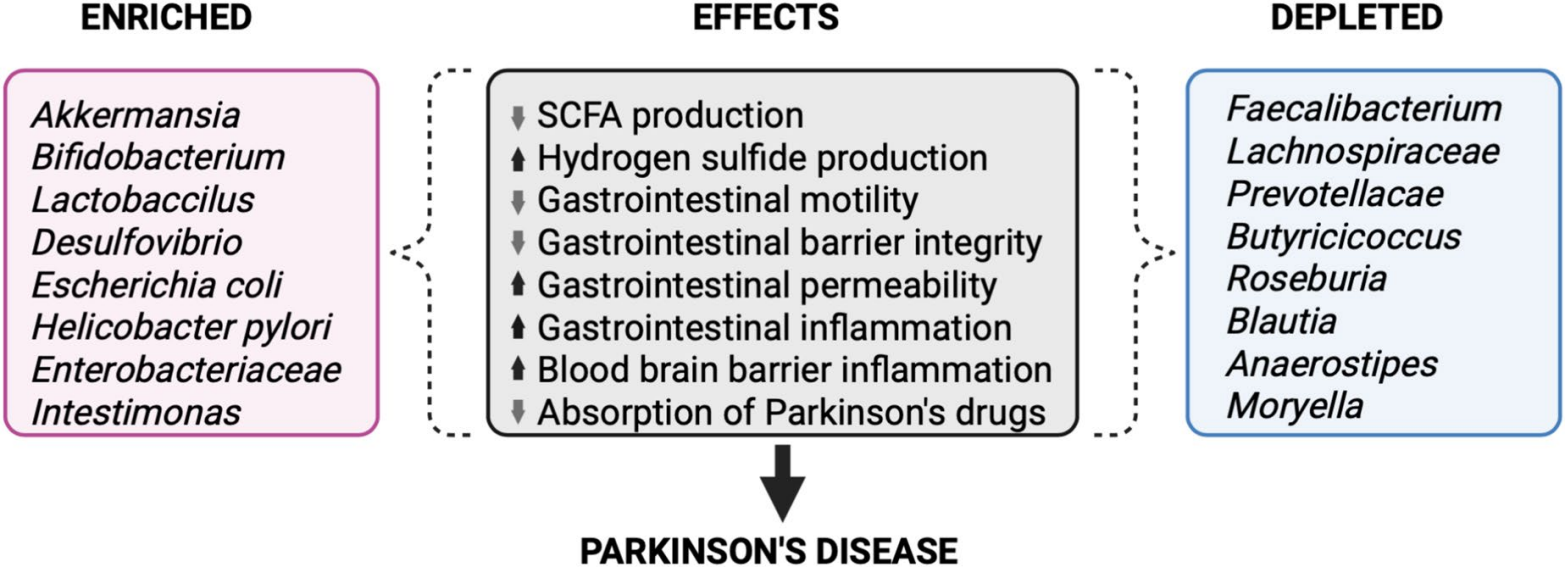
Summary of the altered bacterial taxa and gastrointestinal properties observed in PD. In summary, there is an increase in pro-inflammatory and hydrogen sulfide producing bacteria, coupled with a decrease in anti-inflammatory and SCFA producing species. This drives a series of localized gastrointestinal and systemic changes seen in prodromal PD that enables the later development of clinically recognizable disease (Created with BioRender.com)

There are multiple conflicting reports on how the gut microbiota differs in individuals with PD versus healthy controls. The findings from multiple individual studies, meta-analyses, and systematic reviews using 16S rRNA investigations of the microbiome in PD are summarized in Fig. 2 [95–103]. These metagenomic studies are subject to several limitations and biases which likely accounts for the significant heterogeneity in their findings. There is geographical and environmental variation between cohorts; the included studies predominantly focus on American, European, and Chinese populations which limits the translatability of their findings for global PD populations. Environmental covariates like diet, exercise, and smoking were often not adequately controlled for, which might explain why disease status commonly accounted for a limited proportion of data variability. When covariates were properly accounted for, subgroup analyses were often underpowered. Many commonly prescribed medications, such as proton pump inhibitors, antidepressants, anticholinergics, and laxatives are known to significantly alter the gut microbiome independently from disease [104]. Together these confounders might explain why many metagenomic studies report small or modest effect sizes despite achieving statistical significance. Large variations in the sample sizes are common, ranging from 38 in individual studies to 1269 in meta-analyses. Therefore, many metagenomic studies are not sufficiently powered to detect subtle microbiome differences when correcting for multiple comparisons, the practice of which differs largely between studies. Moreover, the field lacks a priori power calculations. Future studies should seek to justify their sample sizes in relation to expected effect sizes or variance of components, considering necessary samples sizes to adequately control for confounders, and this process should be explicitly reported. In addition, particularly in the context of this review, there is little to no account for the duration of PD in metagenomic studies, hence any microbiome evolution over the course of PD progression will be missed. This dilutes the resolving power of metagenomic studies when the PD microbiome is treated as a single and static entity.

There are also limitations in the use of 16S techniques for identifying bacterial taxa. This classically involves analysis of the V3–V4 hypervariable region of 16S rRNA from fecal samples, a technique not sensitive enough to allow analysis on the species or strain level. One solution is for future studies to use shotgun sequencing of entire bacterial genomes, as some recent studies have moved towards [101], although such measures will be resource and time intensive. Significant variability exists between studies in the bioinformatic techniques used for higher level taxonomic classifications at the level of the genera or family. This comes in part from variation in the reference database and classification systems used across different studies, such as specific amplicon region choices within the V3–V4 region. There is significant heterogeneity in normalization and compositional data analysis, with multiple different techniques used across studies to transform the data for statistical analysis. Future studies should seek to standardize this protocol, although this is difficult when a gold standard approach has not yet emerged. Recent gut microbiome studies are increasingly incorporating functional profiling that aims to predict or directly measure the metabolic and physiological roles of the gut microbiome. Functional profiling often utilizes computational, machine learning, and artificial intelligence algorithms to move beyond p-values and differential abundance into the realm of pattern recognition and integrative prediction. Such techniques are often troubled by overfitting in their models, confirmation biases arising from training the model on and making predictions about the same dataset, and difficulty in making causal inferences. Since functional profiling is so novel the relevant strengths and limitations are not yet clearly delineated; the potential value of these techniques for the future of gut microbiome bioinformatics in early and prodromal PD remains to be determined.

Unpacking the relationship between an altered abundance of microbial species and PD is complex. The abundance of certain gut microbial species is associated with parkinsonian motor symptoms, such as *Enterobacteriaceae* levels which are positively correlated with the severity of postural instability and gait disturbances [105]. *Akkermansia* is consistently shown to be enriched in the gut microbiome of PD patients yet helpfully highlights the non-linear relationship with PD pathology that is commonly seen in gut microbiome research. *Akkermansia* is a mucin-degrading bacterium and investigations on mice have found that *Akkermansia* can deplete the intestinal mucosal layer, decrease the number of goblet cells, produce drier stools, slow colonic transit, and impair intestinal barrier function [106–108]. This could contribute to a leaky gut lumen which drives local and systemic inflammation that drives PD pathogenesis. Multiple strains of *Akkermansia* can co-exist in the gut and host-specific responses can be strain-specific. For example, different strains of *Akkermansia* have been shown to have opposing effects on SCFA production and the differentiation of regulatory T cells which modulate immune responses [109]. Therefore, an increased total abundance of *Akkermansia* in PD can have both disease-promoting and disease-protective effects on gut luminal permeability and local inflammatory modulators, and this relationship is true for many other gut microbial species linked to PD. Many individuals with PD are treated with drugs such as anti-depressants and catechol-O-methyltransferase (COMT) inhibitors which can alter the gut microbiome, including the levels *Akkermansia* [104]. In a similar vein, *H. pylori* infection, associated with PD risk [110, 111], is thought to contribute to motor dysfunction at late stages by reducing levodopa bioavailability, and at early stages through induction of local and systemic inflammation. For example, *H. pylori* induced cytotoxins have been shown to disrupt microglia activity, blood–brain barrier integrity, and trigger neuronal injury and apoptosis [112]. This could create an inflammatory environment that promotes α-synuclein expression, misfolding, and aggregation which subsequently triggers further gut dysbiosis, inflammation, and luminal hyperpermeability initiating a viscous positive feedback cycle [113–117]. This serves as an example of how pro-inflammatory gut microbial species could trigger PD pathogenesis, although future studies are required to demonstrate this on a molecular and mechanistic level.

There is evidence for α-synuclein misfolding and aggregation being promoted by an inflammatory microenvironment that subsequently induces an immune response and further inflammation to create a vicious and disease-promoting positive feedback loop [118–120]. At the molecular level, in vitro data indicate that SCFAs might contribute to α-synuclein pathogenesis. SCFAs, especially butyrate, are used by epithelial cells in the colon as an energy source and a modulator of tight junctions between adjacent epithelial cells [121]. Butyrate-producing bacteria such as *Butyricicoccus* have recognized anti-inflammatory roles and are negatively correlated with plasma levels of inflammatory markers, including interleukin-6, interleukin-8, and C-reactive protein (CRP) [122]. Locally, depletion of SCFA producing bacteria could disrupt the integrity of the intestinal barrier and promote an enteric immune response. SCFAs are shown to modulate activation of the NLRP3 inflammasome via GPR43 activation and modulation of autophagy pathways via autophagy genes including Atg5 and the PI3K/Akt/mTOR pathway. [123, 124]. Together, these processes could destabilize α-synuclein, impair autophagic clearance of α-synuclein, and lead to compensatory stress that disrupts proteostasis and drives α-synuclein misfolding and aggregation. Despite this, the precise molecular mechanisms by which SCFAs and other gut bacterial metabolites alter α-synuclein misfolding and aggregation remain elusive and are yet to be demonstrated in vivo. Systemically, SCFAs could lead to inflammation, disturbed endocrine signaling, and reduced neuronal survival, potentially involving dopaminergic cell death through altered histone deacetylase inhibition [125–127]. Some studies have found no difference in SCFA levels between individuals with RBD compared to controls [128, 129], and one study reported no significant association between the non-motor features of prodromal PD and microbiome changes [130]. One explanation suggested by various authors is that SCFAs only rise during the late stages of prodromal PD and that this is responsible for phenoconversion from RBD to PD with fulminant motor symptoms. Elucidating whether distinct gut microbiome signatures like SCFAs are indicative of prodromal to PD phenoconversion requires future longitudinal studies which can unpick the molecular progression from RBD to early and late stages of PD, at least for body-first subtypes.

Gut microbiome changes have been seen in early and prodromal PD. In support, one study identified depletion of *Lachnospira* and *Butyricicoccus* in prodromal RBD and first-degree relatives of RBD patients [131, 132]. The depletion of *Butyricicoccus* and *Faecalobacteria* have been shown to be specific to PD patients with RBD rather than those without – namely, it suggests that brain- and body-first PD subtypes could have distinct microbiome signatures [133]. Moreover, there was a negative correlation between the levels of these two bacterial species and symptom severity scores for RBD. The authors have proposed that a decreased abundance of *Butyricicoccus* and *Faecalibacterium* might be potential hallmarks of phenoconversion of RBD to PD (Zhang et al. 2024). This claim requires further validation from longitudinal metagenomic studies of the gut microbiome in early PD and RBD. One example of an approach that provided temporal insight into the progression of the gut microbiome at different stages of PD comes from a nested case–control study utilizing two large epidemiological cohorts. This study showed that a microbiome-based classifier had moderate accuracy in discriminating and identifying prodromal and early PD patients according to gut microbiome profile [134]. A separate cross-sectional shotgun metagenomic study compared brain- and body-first PD gut microbiomes according to RBD status and cardiac MIGB uptake [135]. In body-first PD, there was a greater abundance of *E. coli* and *Akkermansia*, and decreased abundance of SCFA-producing bacteria, corroborated by other studies into the prodromal gut microbiome in PD. These differences were associated with a higher abundance of microbial genes associated with curli protein biosynthesis, the significance of which is discussed in more detail below. The most robust and comprehensive account of the gut microbiota in PD, RBD, and age-matched controls comes from Huang et al. [136]. The group performed 16S rRNA analysis of fecal samples and predicted the putative functional metabolic profile of the gut microbiota from RBD and PD patients using a computational machine learning algorithm. The group identified that PD patients display increased fatty acid metabolism, reduced SCFA levels, and decreased synthesis of preQ0 and vitamin B12. Overall, the gut microbiota profile of prodromal and early PD remains poorly defined. Future research into this area should utilize longitudinal RBD and PD cohorts with the aim of identifying the signatures of phenoconversion across microbial, molecular, and clinical levels.

Another question that remains unanswered is where does the pathological α-synuclein in the gut originate? Some studies have suggested that the microbial amyloid protein curli, often produced by *E. coli,* could act as a template for α-synuclein aggregation in the gut [86]. Exposure to curli in the gastrointestinal tract has been shown to increase inflammation and α-synuclein aggregation in the gut and brains of α-synuclein overexpressing mice and aged rats and this is associated with parkinsonian motor deficits [87]. Gastrointestinal enteroendocrine cells that connect directly to the gut lumen and local neurones, and submucosal neurones which synapse micrometers from the gut lumen, could also be sources of α-synuclein [137]. Both cell types naturally express α-synuclein and offer a theoretical source for early pathological α-synuclein aggregation and spread to the brain via the vagus nerve [9, 137]. Following exposure to bacterial metabolites such as lipopolysaccharide (LPS), intracellular levels of α-synuclein in enteroendocrine cells have been shown to increase, suggesting that under specific microbial conditions enteroendocrine cells could be the initial source of PD pathogenesis [138]. It remains unclear how early α-synuclein would spread to the rest of the body from enteroendocrine cells. Dysfunction in the bidirectional interactions between enteroendocrine cells and the hormone glucagon-like peptide-1 (GLP-1) that they secrete has been linked to PD pathogenesis, potentially involving links to SCFA metabolism [139, 140]. There is increasing evidence from animal studies and clinical trials to suggest that GLP-1 receptor agonists could be beneficial in the treatment of PD, and one study using a prebiotic supplement that enhanced SCFA levels showed an increase GLP-1 levels in mouse models [141–144]. However, detailed discussion of these therapies is beyond the scope of this review and is discussed in more detail elsewhere [145]. In addition, *E. coli* is a crucial gut bacterial species that carries out nitrate respiration to produce nitrite and reactive oxygen species, capable of increasing oxidative stress in nearby host cells, particularly enteroendocrine cells expressing α-synuclein [146]. Oxidative stress promotes dopamine oxidation into reactive quinones that can react with α-synuclein to create pathogenic oligomeric forms, inhibit α-synuclein clearance, and initiate pathology at the molecular level [147, 148]. Future studies using animal models of prodromal PD, such RBD, could help to identify where early pathological α-synuclein aggregates originate; and building on this, these studies could pinpoint which host factors such as genetics, ageing, and gut metabolites facilitate the sequence of molecular events that is crucial to phenoconversion.

It is often unclear whether gut microbiota dysbiosis is causal, contributory, or compensatory to PD. If certain changes are compensatory and protective, their therapeutic ‘correction’ may be detrimental. Resolving current gaps in understanding will require a shift from retrospective case–control studies of the microbiome in PD to prospective cohort studies and randomized controlled trials. Future cohort studies could utilize RBD-positive patients as a group at high risk of developing PD. Here, microbiota analyses should include 16S rRNA metagenomics and shotgun sequencing where possible, alongside investigation of non-bacterial microbes, including archaea, viruses, and fungi. These hypothetical studies should be integrated with multi-omic investigations (including metabolomics, transcriptomics, and proteomics) which can draw inferences into the functional biochemical changes in the microbiome that drive PD pathogenesis. This is necessary because healthy individuals display highly divergent gut microbiomes while retaining similar functional capacities [149], hence further work is needed to understand functional changes in the gut-microbiome, interactions between microbes, and hot-microbiome interactions [150]. We must move beyond a unidimensional harmful versus beneficial view of the gut microbiome to one which appreciates the interactions of complex microbial communities and host-microorganism interactions. These advances could support personalized diagnoses, prognostication, and treatments based on the gut microbiome at the prodromal stage.

## A gut-first model of PD opens new avenues for treatment

Existing pharmacological treatments for PD could be improved through an appreciation of the gut microbiome [151, 152]. For example, PD patients develop motor fluctuations of the wearing-off type with continued levodopa use due to increased drug breakdown by peripheral COMT, such as in the gut–COMT inhibitors are a useful adjuvant therapy in PD and increase the time for which levodopa improves clinical symptoms [153]. The gut microbiome has a profound impact on drug metabolism, inter-individual drug responsiveness, and drug efficacy with time [154]. Tyrosine decarboxylase (converts levodopa to dopamine in the intestine), mainly produced by *Enterococcus* (enriched in the PD microbiome), is negatively correlated with plasma levodopa levels [155]. This suggests that microbiota dysbiosis in PD could reduce the availability of levodopa [156, 157]. Similarly, enrichment of gastrointestinal *H. pylori* in PD has been postulated to increase local acidity and reduce levodopa absorption [158]. As such, existing PD treatments could be improved through antibiotic therapies which target gastrointestinal species like *Enterococcus* and *H. pylori*, as reviewed in Table 1.

**Table 1:** Synthesis of evidence for antibiotics, prebiotics, and probiotics for treating PD

Biotic type	Function	Evidence
Antibiotic	Used for killing a specific species of harmful gastrointestinal bacteria	Antibiotic treatment of PD has received most investigation for *H. pylori* eradication therapy. One small study showed that antibiotic mediated *H. pylori* eradication improved levodopa pharmacokinetics [158]. However, a larger study found no difference in levodopa pharmacokinetics in patients with PD based on whether they were *H. pylori*-positive or negative [159]. Several small, open-label studies have identified improvements in motor symptoms after *H. pylori* eradication. In contrast, a randomized, double-blind, placebo-controlled clinical trial in 2020 found no improvements in motor symptoms after *H. pylori* eradication at 12- or 52-week follow ups [160]
Prebiotic	Substrates that are not absorbed or digested by the host, but instead selectively promote the metabolism and proliferation of beneficial bacteria in the body. They are predominantly non-digestible carbohydrates and SCFAs that are produced from their fermentation	In 2017, Perez-Pardo et al. introduced prebiotic fibers to rotenone-induced PD mouse models. This improved rotenone-induced delayed intestinal transit times and reduced rotenone-induced α-synuclein overexpression in the colon [161]. The prebiotic intervention partially alleviated motor and non-motor problems and restored levels of striatal dopamine transporters in these mice, indicating neurorestorative properties. In another study, researchers showed that a diet rich in insoluble fibers (a common prebiotic food source) improved constipation, plasma levodopa bioavailability, and motor function in humans with PD [162]. Another similar population study in humans found that resistant starch (a prebiotic) improved motor symptoms and increased fecal butyrate (SCFA) levels in individuals with PD [163]. However, none of the studies in humans produced statistically significant changes in gut microbiota composition, leaving a crucial gap between clinical findings and mechanistic explanation
Probiotic	Probiotics are live micro-organisms that confer health benefits to the host by improving the balance of the gut microflora. Probiotics inhibit pathogen colonization, exert anti-inflammatory and immunomodulatory effects, and improve gut luminal integrity. There is large heterogeneity in the species used for probiotics	The use of fermented milk as a probiotic (including various strains of *Lactobacillus*, *Bifidobacterium*, and *Enterococcus*) in once daily formulation to treat constipation in PD has been shown to improve questionnaire-based assessments of bowel frequency and stool consistency [164, 165]. Several other RCTs have consistently shown probiotics can improve constipation in PD patients [166–169]. This intervention also reduces CRP and glutathione levels on blood tests (a proxy for inflammation). Other studies which use *Enterococcus* as a probiotic have found negative functional effects, perhaps mediated by the potent levodopa degradation capacity of *Enterococcus* [157]. A recent meta-analysis in 2022 of 12 pro-biotic studies for treating constipation in PD identified increased stool frequency, reduced laxative use, and a reduced severity of motor symptoms [170]. Despite this, there was significant heterogeneity in the individual studies assessed in this meta-analysis: it included studies from a range of countries, with different environmental factors, and using a variety of different types and doses of probiotics. Hence it is unclear how to use these results to guide future practice in probiotic prescribing for PD and prodromal PD, although it does offer hope for early gut-based therapeutic interventions

Antibiotics typically broadly deplete the numbers of many gut microbes with little discrimination between beneficial and harmful species. Recent research in other non-PD conditions has highlighted the potential transferability of targeted approaches like engineered symbiotic bacteria, CRISPR-Cas9 technologies, and bacteriophages for targeting specific bacterial populations [171–173]. Such interventions will have to overcome several safety and logistical hurdles before they are used widely in clinical trials for PD treatment, although this could be possible in the next few years. Moreover, even if specific bacterial species can be depleted, there is a separate problem that comes with highly targeted therapies: depleting a single bacterial species is often not capable of modulating the complex gut-host microbial network in a clinically meaningful or reproducible manner. Future studies of antibiotic therapies in the PD and prodromal PD microbiome should seek to identify how best to target the specific species driving PD pathology while producing a large enough effect to achieve clinically significant results. As identified in Table 1, the current value of antibiotic therapy for treating PD is largely biased towards studies from H. pylori eradication, and therefore, misses crucial detail about the potential role of targeting other bacterial species.

Alongside antibiotics, pre- and pro-biotics offer a route to manipulate the microbiome in prodromal PD [174–176], as assessed in Table 1. These interventions are shown to reverse PD pathology and symptoms in animal models, but these findings have not been reliably replicated in human studies, particularly for motor symptoms. There is less evidence from human studies for pre- and pro-biotic interventions compared to antibiotic treatments. Even when symptom improvements in PD are seen following pre- and pro-biotic treatments, this often does not map onto recognizable changes in the gut microbiome (highlighted in Table 1), underscoring the significance of these clinical findings. There is limited evidence of how pre- and pro-biotics influence gut microbiome composition and functionality, with only limited evidence from a few open-label studies to suggest that pre- and pro-biotics can increase the abundance of SCFA-producing bacteria, reduce the abundance of pro-inflammatory bacteria, and reduce levels of intestinal inflammatory markers like calprotectin [163, 177]. In the context of the gut-first model of PD considered in this review, all human studies of biotic interventions have lacked clear causality arguments in their hypotheses and experimental design, whereby improvements in motor symptoms are often seen as a secondary outcome and are typically not complemented with investigations of the gut microbiome profile. Future randomized controlled trials that compare pre- pro- and anti-biotic preparations compared to placebos over time together with fecal microbiome taxonomic investigations are required to provide robust evidence for clinically using these treatments in the context of PD. Paradoxically, various bacterial strains used as probiotics—such as *Lactobacillus* and *Enterococci*—are the same strains that are enriched in PD. This reinforces the notion that microbiota communities can only be understood as a functional unit rather than a sum of their individual components. This creates a complicated chicken-and-egg problem whereby it is unclear how to positively modulate the microbiome with antibiotics, prebiotics, and postbiotics when it is unclear what a healthy and unhealthy microbiome looks like in relation to PD. The longitudinal and multi-omic investigations of the prodromal PD microbiome discussed earlier will help to identify common features of the gut microbiome that drive PD progression. This could aid the development of more targeted ‘–biotic’ preparations which could have greater success in clinical trials, at least for patients with RBD and presumed body-first PD.

Diet offers an attractive avenue to ameliorate PD progression at early or prodromal stages [178]. Small, population-based studies have found limited support for ketogenic, low-fat, and vegetarian diets, although it is currently not possible to discern whether this is simply because populations eating these diets are healthier at baseline [179–181]. One recent study employed 16S rRNA sequencing and predictive metagenomics to identify that higher fiber intake was associated with enriched anti-inflammatory species such as *Butyricicoccus*, while high sugar intake was associated with enriched pro-inflammatory species such as *Klebsiella* [182]. The consumption of fiber and sugar has been linked to key gut pathways implicated in PD pathogenesis, including microbial diversity, SCFA synthesis, and intestinal luminal integrity [183, 184]. Since individual macro- or micronutrients are rarely consumed in isolation, it is important to also consider the effects of broader dietary patterns. A meta-analysis and systematic review of 24 studies investigating the role of dietary supplements, foods, and dietary patterns in PD found that dietary supplements or food intervention did not affect the total Unified Parkinson Disease Rating Scale (UPDRS) score, or 6-min walk test. However, this study identified that dietary patterns intervention, namely Mediterranean, low-fat, low-protein, and ketogenic diets, produced clinically meaningful improvements in motor and non-motor symptoms [185]. Moreover, the Mediterranean diet has been associated with a reduced risk of prodromal PD among older adults in Greece [186]. The case–control studies are limited by selection diet recall bias, while prospective dietary cohort studies are limited by the requirement for extremely large populations and the issue of reverse causality, whereby individuals change their diets due to the early signs of the disease [9]. Because of infinite confounders such as medication history, geographical variation in food availability and quality, and host-specific factors, there is large interindividual variation between the responses to dietary interventions for PD, as seen for other chronic conditions [187]. Therefore, further human research is required before dietary recommendations can be made as a prophylactic treatment for prodromal or early PD.

Several lifestyle and environmental factors have been linked with PD and offer novel therapeutic avenues. Smoking and caffeine have been shown to be protective against PD [23, 188]. Smoking reduces gut inflammation, decreases luminal epithelial permeability, and decreases the abundance of *Enterobacteriaceae* [189, 190]. Caffeine promotes gastrointestinal permeability and decreases the abundance of species such as *E. coli*, helping to counteract constipation [191]. Despite this, recommending smoking and caffeine consumption should not be advised as a method of protecting against PD, not to mention the other negative health effects caused by smoking. There is an increasing array of environmental factors being linked with PD and the microbiome, including pesticides like rotenone, paraquat, and glyphosate [192]. Pesticides are thought to contribute to central and peripheral neurotoxicity, induce gut microbiome dysbiosis, and drive host-microbiome toxicity [193, 194]. Proposed mechanisms for this pathology include a combination of inflammatory dysregulation and metabolic alterations, such as insulin resistance and ghrelin-dysregulation that produces neurodegeneration [195, 196]. Strategies to reduce the pesticide disease burden include reducing exposure such as through replacement with biopesticides and genetically modified organisms, greater monitoring for pesticide contamination in sources like drinking water and food, and adoption of protective techniques to include simple measures like hand washing with soap [197, 198]. Due to limited evidence, this review will not consider other lifestyle, environmental, and other novel means to modulate the gut microbiome in prodromal PD such as small molecule drugs, biologics, engineered symbiotic bacteria, and microbiota-derived proteins and metabolites [199].

Fecal microbiota transplants (FMTs) could be used as a safe and efficacious method to manipulate the gut microbiome and treat PD and is licensed for use in many countries for treating *Clostridium difficile* infections [200, 201]. FMTs from wild-type to drug-induced PD mice has been shown to reduce gut microbiota dysbiosis, alleviate motor impairments, and increase striatal dopamine levels [202]. Follow-up targeted assays revealed that these effects were mediated by suppressing neuroinflammation and reducing toll-like receptor 4 and tumor necrosis-α signaling [203]. Other FMT studies in mice have corroborated these findings, suggesting that FMT can reduce constipation and tremors while increasing levels of protective bacterial species such as *Prevotella* [204]. In a clinical context, FMT has been used to successfully treat constipation in a small population of PD patients where donor fecal microbiota from healthy individuals was administered endoscopically without any serious side effects [205]. There are currently four RCTs with available evidence for the role of FMT in treating PD [206–209]. Three of these reported changes in global or motor function, and of these two reported significant improvement UPDRS PD symptom scores at 12 weeks and 12 months, respectively. In these studies, the clinical findings were associated with a shift toward anti-inflammatory microbial species. One study reported no changes in motor function at 6 months post-FMT, although here the treatment group required smaller dose increases than placebo. In this study, only 3% of the treatment group were reported to show a change from dysbiotic to non-dysbiotic status, compared to 33% in the placebo arm, suggesting that the FMT treatment failed to produce a significant change in the gut microbiome. The heterogeneity in findings for these clinical studies could be explained by the following study limitations: very small sample sizes; differences in the choice of donors and the selection of recipients; and the use of various placebo and FMT protocols that involved differences in preparation methods and administration routes. Compared to the other treatment modalities outlined in this review, FMT has the strongest mechanistic support from animal models of PD. Together with the rapidly growing utility of FMT for treating several other conditions in recent years [210, 211], FMT offers the greatest immediate hope for producing clinically meaningful therapeutic modulation of the gut microbiome in prodromal and early PD.

One exciting notion is that FMT and other microbiome-altering interventions could be used in the future to prevent phenoconversion of prodromal PD. Testing this hypothesis could come, for example, from using FMT to treat the gastrointestinal symptoms experienced by many individuals with RBD, many of whom will have prodromal PD. Success would be measured by whether the intervention can reduce the 6.3% of individuals with RBD that phenoconvert to overt α-synucleinopathies each year, mostly PD. Future clinical trials are necessary to investigate this hypothesis and should be complemented with the metagenomic and multi-omic investigations of the gut microbiota outlined earlier to link clinical outcomes with mechanistic explanations. One difficulty that could be encountered is whether the prodromal phase represents the right time window for intervention, especially given that the prodromal phase itself is a dynamic process capturing multiple stages of pathological progression. Utilizing retrospective and prospective longitudinal patient cohorts including control, RBD, and PD patients with varying disease durations could capture the temporal spectrum of disease progression and will offer a good starting point for identifying the right time for therapeutic intervention. However, the inter-individual heterogeneity of RBD and PD pathophysiology and progression will complicate this matter. We suggest that early clinical interventions should initially focus on FMT as the intervention-type most likely to produce meaningful effect sizes that can be detected through both microbiome alterations and clinical symptoms. If proven to be successful, more targeted approaches like pre- pro- and anti-biotics could be trialed, although these are likely to require larger sample sizes and a better understanding of gut microbiota dysbiosis in driving PD to refine these therapies in the first place. Lifestyle and environmental interventions that include dietary changes and pesticide exposure currently lack evidence to make specific recommendations; simple and general measures like eating a healthy diet high in fruit and vegetables and ensuring good hand washing and hygiene might offer a protective value against PD, alongside being good measures for overall health across virtually every measurable metric. To this avail, it remains to be determined whether PD can be prevented in a subset of patients with gut-based pathology through gut-based treatments.

## Conclusions

PD is canonically understood, investigated, and treated as a disorder of dopaminergic signaling in the brainstem. This review considers a return to a historical appreciation of PD as a disorder originating from the gut. PD could be mediated by gut microbiota dysbiosis in prodromal states before the onset of motor symptoms, at least in a subset of patients with body-first PD who often display RBD. It seems likely that a combination of gastrointestinal constipation, inflammation, impaired luminal integrity, and disturbed fatty-acid metabolism could mediate this effect, potentially seeding prionoid gut-to-brain propagation of α-synuclein via the vagus nerve. However, the current evidence to support these claims remains largely speculative.

Antibiotics, prebiotics, probiotics, and FMT are gut microbiome altering treatments that can successfully treat the non-motor symptoms of PD, with some support for improving motor symptoms. Gaps in our current understanding can be overcome by prospective, interventional, and longitudinal studies in RBD only, RBD-positive PD, and control participants that can outline the timeline of gut dysbiosis in prodromal PD and how to target therapeutic interventions. These investigations should be coupled with experiments to resolve outstanding questions on the functional effects of microbiota dysbiosis using a range of multi-omic techniques, targeted manipulations in animal models, and detailed analysis of confounding factors such as age, diet, and medication use. This could aid the development of personalized microbiota-based identification and treatment for prodromal PD. Utilizing the gut–brain axis could enable early intervention to mitigate phenoconversion and reduce the global disease burden of PD.
